# Effect of Isoniazid Preventive Therapy on the Incidence of Tuberculosis among Seropositive Children Attending HIV/AIDS Care in Two General Hospitals, Northwest Ethiopia, 2021

**DOI:** 10.1155/2021/9996953

**Published:** 2021-08-25

**Authors:** Fassikaw Kebede, Birhanu Kebede, Tsehay Kebede, Melaku Agmasu

**Affiliations:** ^1^Department of Epidemiology and Biostatistics, School of Public Health, College of Health Science, Woldia University, Woldia, Ethiopia; ^2^Agricultural Extension and Family Health Team Leader, Pawe Woreda Metekel Zone, Pawe, Ethiopia; ^3^Department of Geography and Environmental Study College of Social Science, Bahir Dare University, Bahir Dar, Ethiopia; ^4^Department of Psychology College of Social Science, Injibara University, Injibara, Ethiopia

## Abstract

The human immune deficiency virus (HIV) is the strongest risk factor for the incidence of tuberculosis (TB) by way of reactivation of latent or new infection. The provision of isoniazid preventive therapy (IPT) is one of the public health interventions for the prevention of TB. To date, there have been limited clinical data regarding the effectiveness of isoniazid preventive therapy (IPT) on TB incidence. This study aimed to assess the effect of isoniazid preventive therapy on the incidence of tuberculosis for seropositive children in Northwest Ethiopia. *Methods*. A facility-based retrospective follow-up was employed for reviewing 421 files from 1 January 2015 up to 30 December 2019. EpiData version 3.2 and Stata/14 software were used for data entry and analysis, respectively. Categorical variables at bivariable Cox regression were assessed for candidates transferred at *P* value <0.25 for multivariable Cox regression to claiming predictors associated with TB incidence rate at 95% CI at *P* < 0.005. *Result*. The overall incidence of TB was found to be 4.99 cases per 100 person-years at 95% CI (3.89–6.53). Missed IPT (AHR = 7.45, 95% CI: 2.96, 18.74, *P* < 0.001), missed cotrimoxazole preventive therapy (CPT) (AHR = 2.4, 95% CI: 1.84–4.74, *P* < 0.022), age ≥ 11 years (AHR = 4.2, 95% CI: 1.04–7.03, *P* < 0.048), MUAC ≤ 11.5 cm (AHR = 4.36, 95% CI: 1.97–9.97, *P* < 0.001), WHO stages III and IV (AHR = 2.04, 95% CI: 1.12–3.74, *P* < 0.022), and CD4 count ≤100 cells/*μ*l (AHR = 3.96, 95% CI: 1.52–10.34, *P* < 0.005) were significantly associated with TB incidence. *Conclusion*. Concomitant administration of ART with IPT had demoted more than ninety-six percent of new TB incidences for this report. Undertaking in-depth TB screening and frequent follow-up among all these children is critical in order to prevent and control tuberculosis.

## 1. Introduction

The human immune deficiency virus (HIV) is the strongest risk factor for latent or new infection of tuberculosis (TB) through target reduction of CD4 T-lymphocytes and cellular immune function [1]. Despite this fact, both (TB/HIV) coinfections are “bidirectional and comrade each other, in which HIV sustains the progression of latent tuberculosis bacilli into active TB, while tuberculosis accelerates the progression of HIV disease to its advanced clinical stage [2, 3]. The waning of the immune system increased *Mycobacterium tuberculosis* susceptibility and progression of dormant tuberculosis bacilli to endogenous reactivation of latent incidence in the lung [4]. As the CD4 lymphocyte count declines, the risk of active TB further increases to reach levels of between 15% and 35% per annum risk in patients with CD4 cell counts of less than 200 cells/*μ*l [4]. TB is the leading lethal immune-suppressing infection and responsible for one-third of TB/HIV-associated death for children living with HIV infection [1, 5].

Globally, an estimated 1.2 million cases of tuberculosis occurred in seropositive patients, of which 1.0 million cases occurred among children (≤15 years) living with HIV [1, 3]. World Health Organization (WHO) TB/HIV program recommends intervention pillar strategy of intensified case finding (ICF), isoniazid preventive therapy (IPT), and infection control (IC), rather than early initiation of antiretroviral therapy (ART) for risk attenuations rate of tuberculosis during successive cohort [6]. However, ART alone is not adequate enough in reducing tuberculosis risk in HIV-infected individuals; hence, the implementation of other TB-specific interventions to further reduce the risk of TB in HIV-infected individuals is an imminent and inevitable task [7]. Global TB report of 2018 narrated that 57% of HIV-positive TB cases were on ART, while 520,000 were reported to have received isoniazid preventive therapy (IPT) [2, 3]. Ethiopia ranks seventh amongst the world's 22 high TB burden countries and TB is the second leading cause of hospital death [3]. In fact, 79% of HIV-infected individuals were screened for active TB, of whom 15% had TB/HIV coinfection [8].

Multiple studies have demonstrated the effectiveness of IPT in adults [3–5, 9–13]. Recent evidence has shown that the combined or concomitant use of IPT and HAART among HIV-infected patients significantly reduces the incidence of TB up to 97% irrespective of CD4 count [14]. Nevertheless, adherence to IPT completion rate is still a despairing issue. Notable factors like drug stock-out, a distance of health facilities, and drug side effects were some of the hindrances for recommended compilation rate [15, 16]. There is a paucity of data for protecting and effective prevention of IPT on the incidence of active TB for children living with HIV during successive time cohorts. This study aimed to assess the effect of isoniazid preventive therapy on the incidence of tuberculosis for seropositive children in Northwest Ethiopia.

## 2. Methods

### 2.1. Study Design, Area, and Populations

**Figure 1:**
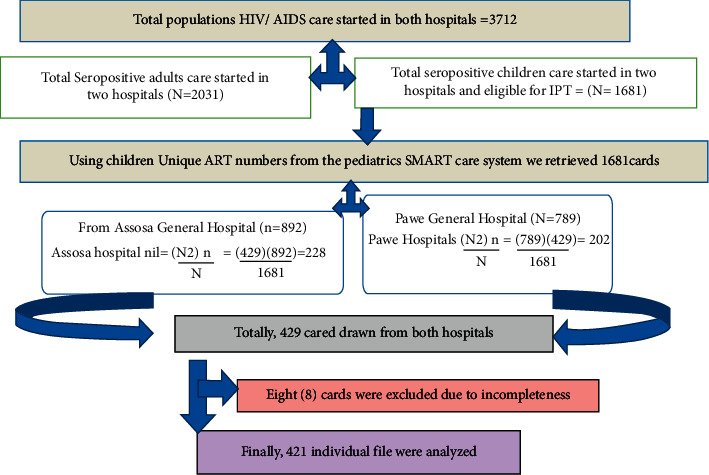
Schematic presentations of sampling procedures from Assosa and Pawe Hospitals.

Facility-based retrospective follow-up study was employed among 421 seropositive children from 1 January 2015 up to 30 December 2019 in Assosa and Pawe General Hospitals. Both hospitals were located in Benishangul Gumuz region, northwest of Ethiopia [17], Those hospitals have provided therapeutic and palliative care services for more than 3712 Assosa registered HIV-positive populations [9, 12]. The source population for this study was all seropositive children enrolled in HIV/AIDS care follow-up whose age is ≤ 15 years. Following the time of HIV/AIDS enrollment of the ART care continuum, a total of 1681 seropositive children started antiretroviral therapy (ART) at both Assosa and Pawe General Hospitals (Figure 1).

### 2.2. Inclusion and Exclusion Criteria

All HIV-positive children below 15 years of age and newly enrolled in pediatric chronic HIV care clinic at Assosa and Pawe General Hospitals from September 2006 to August 2010 were included in this study. Those HIV-positive children who started anti-TB treatment at the beginning of the follow-up and those with incomplete baseline information such as CD4 count and hemoglobin (Hgb) level were excluded from the study.

### 2.3. Outcome Ascertainment

In the study setting, TB is diagnosed using chest radiology, fine needle aspiration, and cytology with very high clinical suspicion. When a child is diagnosed with active TB, the treatment is given according to the national TB treatment guideline. The event of this study was the new incidence of TB considered as an event of inters, which is defended as the occurrence of TB in HIV-infected children during the follow-up period at any time after enrollment to pediatrics HIV care clinic. Children who were lost, died, transferred out, or did not develop the events until the last visit were considered censored, whereas variables including age, sex, residence, family size, WHO clinical stage, CD4 count, Hgb, functional status, and nutritional status like stunting, wasting, and underweight were considered as independent variables.

### 2.4. Sample Size Determinations and Sampling Technique

The sample size of this study was calculated based on single population proportion formula using the assumptions of two-sided significant level (*α*) of 5%, *Z* a_½_ value at 95% confidence interval = 1.96, prevalence of IPT taken from previous study = 50.2% [3]. The sample size would be 384 by adding 10% for the incompleteness of data contingency and become 429. From the two hospitals, 1681 children registered for HIV/AIDS care from 2015 to 2020. Based on the source population, the final sample sizes were drawn by computer-generated simple random sampling using unique ART number as a sampling frame from pediatrics SMART care; 227 from Assosa and 202 from Paw Hospitals were drawn, respectively.

### 2.5. Operational Words

Isoinized Preventive Therapy (IPT): a drug prescribed for prevention of endogemouse reactivation of latent incidence of tuberculosis (TB), while after ruling out of active TB symptoms and recommended as a daily dose of 300 mg/day for adults and 10 mg/kg for children at least 6–12 months [18]. The effect of IPT was measured as new occurrence of TB for IPT group times by % and divided for all IPT initiated children.

### 2.6. Data Collection Tools and Quality Control

Data were collected using data abstraction tools (checklist) prepared from Ethiopia's Federal Ministry of Health Pediatrics antiretroviral therapy (ART) follow-up and medical history sheet [19]. Four diploma nurses and two BSc nurses were recruited for data collection and supervision *h*. One day of training was given for data collection and supervision. Before the actual data collection, prepared checklist was pretested on 5% of the original sample size files in another health hospital. The questionnaire was modified based on pretest results. The collected data were first being checked and cleaned for completeness. To ensure the quality checklist, 5% of the final sample size pretest was done and the necessary amendment was incorporated on the checklists before date collection. The collected data were first being checked and cleaned for completeness before data analysis.

### 2.7. Data Analysis

EpiData version 3.2 and Stata/14 software were used for data entry and analysis, respectively. Proportional hazard assumption was checked for each variable and no variable was found with Schoenfeld residual test <0.05. Categorical variables at bivariable Cox regression were assessed for candidates transferred at *P*-value <0.25 for final multivariable Cox regression models, and variables associated with TB incidence in 95% CI at *P* < 0.005 were claimed as the predictor.

## 3. Results

### 3.1. Sociodemographic Characteristics

Four hundred twenty-one (421) seropositive files were included for final analysis. Eight (8) individual cards were excluded due to incompleteness. The overall response rate of the participant was determined as (421/429; 98.25%). The mean age of children was 8 years (SD ± 3.66). Nearly two in every five (165/421; 39.19%) of the study participants were school-age children (6–10 years), whereas more than half (217/421; 51.54% and 219/421; 52.02%) of the study participants were female and were from rural residents, respectively **(**Table 1).

### 3.2. Baseline Clinical and Laboratory Characteristics

Nearly one-third (274/421, 65.08%) of the study participants had hemoglobin ≤ 10 mg/dl, whereas three in every four (75.3%) of the participants had CD4 cell count ≥ 201 cells/mm3. Moreover (316/421) 75.06% of children took cotrimoxazole prophylaxis (CPT). On the contrary, nearly two in every five (168/421, 39.9%) of the participants missed IPT care started. Dealing with the WHO clinical stage of children (128/421) 30.4% and (89/421) 21.14% of the HIV-infected children were found in clinical stages I and III, respectively. Nearly two in every five (164/421, 38.95%) of the participants had at least one episode of opportunistic illness in the past. Of the total 421 participants, 81 (19.24%) children shifted baseline ART regimen. Accordingly, the frequent reason for the change was reported to be toxicity and TB incidence (24/81 and 22/81), respectively. Nutritional status of study participants: at baseline, 29 (6.89%), 33 (7.84%), and 46 (10.93%) of the study participants were severely underweight, stunted, and wasted, respectively (Table 2).

### 3.3. Effect of Isoniazid Preventive Therapy and TB Incidence

Of all study participants, nearly two-thirds of the children developed at least one episode of opportunistic illness in the past, 103 (63.1%) of them were from the non-IPT groups within 10315.63 person-years of observation (PYO), whereas the remaining 61 (36.2%) were among those who took IPT. The median CD4 cell count was found to be 482.6 cells/*μ*l (IQR = 277.5–907.80) for entire cohort groups. During risky observation of children, 52 new tuberculosis cases were reported. Of the total number of TB cases, those in the non-IPT group were found to be 46/52 (88.46%).

### 3.4. Incidence Density Rate (IDR) of TB

The incidence rate of TB cases who did not take isoniazid preventive therapy was 11.5 per 100 person-years (95% CI: 8.42–15.02). Furthermore, 6/52 (9.11%) of new TB cases were from IPT groups; the incidence rate was 1.14 cases per 100 person-years (PY). The overall incidence rate of TB was determined as 4.99 per 100 person-years within 95% CI (3.89–6.53). The chi-square test for the risk of the hazard difference between IPT and non-IPT indicated that TB-free survival probability in IPT user groups was significantly higher than that in groups who do not take IPT (Log-rank test = 55.64, df = 1, *P* < 0.001) (Table 3).

**Figure 2:**
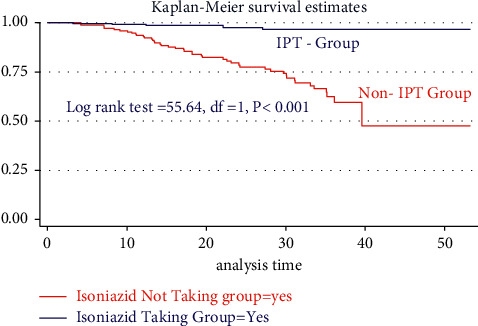
Kaplan–Meier estimate of tuberculosis-free survival probability in the IPT and non-IPT group in two hospitals since 2015–2020.

**Figure 3:**
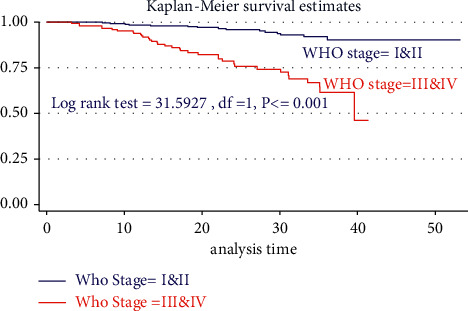
Kaplan–Meier estimate of tuberculosis-free survival probability between WHO clinical stages in two hospitals since 2015–2020.

**Figure 4:**
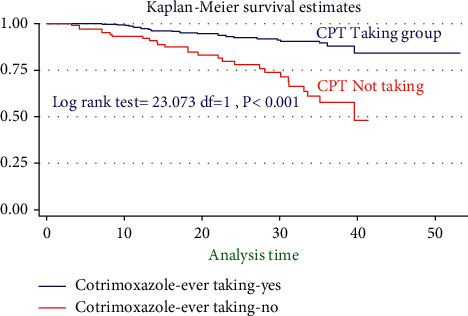
Kaplan survival curve for ever taking and missed status of co-trimoxazole preventive therapy among seropositive children started HIV/AIDS care in two general hospitals from 2015–2020.

**Figure 5:**
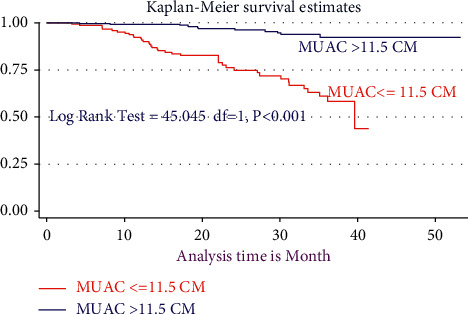
Kaplan–Meier estimate of tuberculosis-free survival probability among MUAC ≤ 11.5 cm and MUAC> 11.5 cm in two hospitals in 2021.

**Figure 6:**
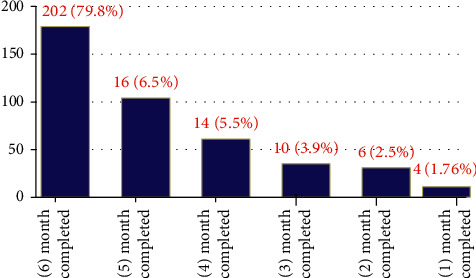
Month of isoniazid preventive therapy full dose completion of seropositive children in two hospitals since 2015–2020.

From a total of 253 (60.10%) children who initiated IPT during 10315.63 person-years of risky observation, merely 201 (47.74%) completed the six-month full course of therapy. The overall incidence of TB for those who took IPT was found to be 1.14 cases per 100 person-years with 95% CI (5.255–23.1) (Figures 2–6).

### 3.5. Predictors for Tuberculosis Incidence

During the bivariable analysis of Cox regression after the proportional hazard assumption (PH-tested) and checked, 16 variables were selected and transferred for multivariable Cox regression at *p* < 0.25. However, after controlling, the confounding 10 variables had built the final adequate models, and six independent variables were associated with the incidence of TB. Therefore, the risk of developing TB among HIV-infected children for seropositive children not initiating CPT was 2.24 times (AHR = 2.4, 95% CI: 1.84, 4.74, *P* < 0.022) higher than counterparts. Moreover, seropositive children aged ≥ 11 years were 4.2 times (AHR = 4.2, 95% CI: 1.04, 7.03, *P* < 0.048) more than those aged ≤ 5 years. Likewise, children who did not initiate IPT were 7.5 times (AHR = 7.45, 95% CI: 2.96, 18.74, *P* > 0.001) more than their counterparts. Also, HIV-infected children who had MUAC ≤ 11.5 cm were 4.4 times (AHR = 4.36, 95% CI: 1.97, 9.97, *P* < 0.001) more than their counterparts. Additionally, seropositive children with WHO stages III and IV were 2.04 times (AHR = 2.04, 95% CI: 1.12, 3.74, *P* < 0.022) more than children with WHO stages I and II. The risk of developing TB among children who had CD4 cell count <100 cells/*μ*l is nearly 4 times (AHR = 3.96, 95% CI: 1.52, 10.34 *P* < 0.005) higher than that of children with CD4 ≥ 200 cells/*μ*l (Table 4).

## 4. Discussion

The report of this research revealed that concomitant administration of IPT with ART significantly reduced the 96.8% incidence rate of active TB. This report had higher results than those found in Central Ethiopia 80% [20], Jimma University 75% [3], Brazil 76% [11], and Indonesia 79% [14]. The Concomitant administration of antiretroviral therapy with IPT markedly effectively reduces the risk for TB incidence in seropositive children, especially when started at early infancy stages [9]. However, in this report, 4 out of 10 seropositive children had still missed initiation of isoniazid preventive therapy. Moreover, the incidence density of TB/HIV-coinfected children in this study was 4.99 (95%CI; 3.89–6.53) per 100 child-years of follow-up. This was slightly consistent with study findings in Northern Ethiopia of 4.2 per 100 child-years [15] and Gondar Hospital of 4.9 per 100 child-years [21]. Such incidence difference might be due to the difference in the follow-up period sample sizes and study setting. Nevertheless, this finding has lower hazards of developing TB incidence as compared to reports in Adama Hospital of 6.03 per 100 person-years [13] and Northwest Ethiopia of 9.6 per 100 person-years [5]. But higher hazards of TB incidence was found as compared to those reported in Debre Markos Hospitals of 2.63 per 100 person-years [22] and Southern Ethiopia of 2.89 per 100 person-years [2]. The difference might be because the study area had a predominant prevalence distribution of TB (>67%) [17]. This might be due to the variation in the type of care provision across different health institutions and different study populations. Regarding predictors for TB, age is the only sociodemographic factor associated with incidence. The hazards of developing TB for age ≥ 11 years were significantly associated with TB incidence. This is similar to results reported in Northern Ethiopia [23], Assosa and Pawe Hospitals [4, 5], Cameron [24], and Asian multicenter cohort of children [25]. This might be due to the failure of immune restoration in chronic carriers of HIV [26] and experiencing high social and environmental interactions among children during this age easily exposed the acquisition of active TB [27]. In fact, WHO recommends isoniazid preventive therapy with a daily dose of 10 mg/kg for at least 6 months for risks to prevent the first episode of TB infection, latent reactivation, and recurrent TB incidence. The finding of this report revealed that missed IPT was significantly associated with TB occurrence. This is comparable with reports in Adama Hospitals [13] and Northern Ethiopia [15]. Concomitant taking of IPT with ART decreases mycobacterium load and puts off reactivation of latent bacillus [28]. Likewise, advanced WHO clinical stages III and IV were significantly associated with TB incidence as compared with the counter group. Findings from a retrospective cohort study in Northwest Ethiopia and Jimma and Gondar Referral Hospitals were similar to our findings [3–5, 15]. HIV wanes the immune system and acceleration of viral replication and is responsible for the sharp reduction of white blood cells and CD4 count [27, 29]. All this ends with a repeated episode of opportunistic infection and a higher risk of developing tuberculosis. On the contrary, CPT is inexpensive and highly effective in reducing morbidity and mortality causes associated with opportunistic infection and prevents load of latent TB lung [20]. However, HIV-infected children in this report who missed CPT were significantly associated with the hazards of developing TB incidence. This finding is comparable with those reported in Gondar Hospital [30] and Adama Hospital [13]. Moreover, the study participants who had MUAC ≤ 11.5 cm were highly associated with TB incidence. This report is similar to the findings of Adama Hospital [13] and Gondar Referral Hospital [31]. According to this study finding, HIV-infected children with acute malnutrition are susceptible to opportunistic infection as compared to those in usual times in Tanzania [32] and Uganda and Zimbabwe [33]. It is not surprising that HIV infection increases nutrient malabsorption and metabolic alterations for critical causes of severe acute malnutrition [34]. In this study, among the determinant factors, having a lower CD4+ count < 100 cells/*μ*l was associated with increased relative hazard for developing TB. The risk of TB shows a higher increase when CD4+ cell counts fall below 350 cells/*μ*l. This result is consistent with cohort studies that show a gradual increase in the risk of TB when CD4+ cell count falls down in South Africa [35]. Besides, a multicenter observational research finding on CD4 count reduction indicated that one episode of opportunistic infection other than TB demoted CD4 count 54–57 cells/*μ*l and led to substantial increment for active TB incidence [34, 36].

## 5. Conclusion

Concomitant administration of ART with IPT had demoted more than ninety-six percent of new TB incidences for this report. Undertaking in-depth TB screening and frequent follow-up among all these children is critical in order to prevent and control tuberculosis.

## 6. Limitations

The retrospective nature of this study design is limited to include other factors that may influence the risk of TB and viral suppression is not recorded continuously since 2015–2020, which has a potential bias on research.

## Figures and Tables

**Table 1:** Baseline sociodemographic characteristics of seropositive children in Assosa and Pawe General Hospitals since 2015–2020 (*N* = 421).

Variables	Categories	Frequency	Percentage
Age of children	≤5 years	94	22.3
	6–10 years	165	39.52
	≥11 years	162	38.48

Sex of children	Male	204	48.46
	Female	217	51.54

Caregivers' religion	Orthodox	189	44.89
	Muslim	86	20.19
	Protestant	110	26.13
	Catholic	36	8.31

Residence	Rural	217	52.3
	Urban	204	48.69

Family size	≤2	135	32.07
	3–4	231	54.87
	5–6	52	12.10
	≥7	4	0.95

Current children status	Living with parents	348	82.1
	Living with relatives	22	5.52
	Orphanage	51	12.16

**Table 2:** Baseline clinical and laboratorial characteristics of seropositive children attending HIV/AIDS care in two general hospitals since 2015–2020 (*N* = 421).

Variables	Category	Total	Percentage
Hemoglobin	<10 mg/dl	147	34.92
	≥10 mg/dl	274	65.08

WHO stages	Stage I	128	30.4
	Stage II	147	34.92
	Stage III	89	21.14
	Stage IV	57	13.54

Functional status	Working	30	72.92
	Ambulatory	72	17.10
	Bedridden	42	9.98

CD4 levels	<100	30	7.13
	101–200	741	17.58
	≥201	317	75.3

ART regimen	4c = AZT+3 TC + NVP	255	60.5
	4d = AZT+3 TC + EFV	64	15.2
	1e = TDF+3 TC + EFV	70	16.62
	4g = TDF+3 TC + NVP	21	2.14
	4a = d4t+3 TC + NVP	9	4.99
	Others	2	0.48

Shift of the regimen	Yes	81	19.2
	No	340	80.76

Reasons for regimen change	Due to toxicity	24/81	29.63
	Due to TB	22/81	27.1
	New drug	5/81	6.17
	Clinical failure	23/81	28.9
	Immunological failure	7/81	8.64

Vaccination status	Fully vaccinated	328	77.91
	Defaulted	33	7.89
	Not started	60	14.25

CPT	Yes	316	75.06
	No	105	24.94

IPT	Yes	253	60.10
	No	168	39.9

Adherence status	Good	239	56.77
	Fair	118	28.03
	Poor	64	15.03

Opportunistic infection	Yes	126	29.93
	No	295	70.70

Follow-up	Alive and on follow-up	361	85.75
	Lost from follow-up	22	5.23
	Transfer out	29	6.88
	Died	9	2.14

MUAC	≤11.5 cm	145	34.44
	>11.5 cm	276	65.56

Underweight	No undernutrition	277	65.80
	Moderate undernutrition	115	27.32
	Severe undernutrition	29	6.89

Stunting	No stunting	284	67.46
	Moderate stunting	104	24.7
	Severe stunting	33	7.84

Wasting status	No wasting	304	72.21
	Moderate wasting	71	16.86
	Severe wasting	46	10.93

**Table 3:** Incidence rate of TB per 100 person-years for IPT and non-IPT groups in two general hospitals since 2015–2020 (*N* = 421).

IPT status	Numbers (%)	New TB case	No. of risk observations (years)	TB incidence per 100 PY (95% CI)
Ever given	253 (60.10)	7	6350.47	1.24 (1.15–3.14)
Missed	168 (39.9)	45	4061.68	11.12 (8.2–14.8)
Overall	—	52	10412.15	4.99 (3.8–6.64)

**Table 4:** Bi-variate and multivariate Cox regressions for incident of tuberculosis among seropositive children in two hospitals of Northwest Ethiopia from 2015–2020 (*N* = 421).

Variables		TB case	Censored	IPT group	Non-IPT	CHR = 95% CI	AHR = 95% CI	*P* < 0.05
Sex	Male	30	174	123	81	1.39 (0.745–2.248	1.2 (0.618–2.53)	0.53
	Female	22	192	130	87	1	1	—

Age of children	< =5 years	2	85	60	34	1	1	—
	6–10 years	12	134	94	71	3.23 (1.24–8.42)	2.214 (0.8–5.9)	0.134
	≥11 years	50	138	99	63	2.8 (1.05–7.49)	4.2 (1.04–7.0)	0.04^*∗*^

Isoniazid (IPT)	Yes	15	243	253	—	1	1	—
	No	49	114	—	168	10.47 (4.68–23.1)	8.567 (3.5–21.5)	0.01^*∗*^

Cotrimoxazole (CPT)	Yes	28	293	198	55	1	1	—
	No	36	64	118	50	3.97 (2.25–6.7)	2.2 (1.11–3.7)^*∗*^	0.022^*∗*^

TB history of contact	Yes	53	82	83	96	2.8 (1.63–4.93)	1.4 (0.75–2.9)	0.26
	No	11	275	72	170	1	1	—

CD4 count	< 100	6	24	16	14	3.99 (1.6–9.77)	3.96 (1.5–10.3)	0.005^*∗*^
	101–200	21	53	38	36	4.67 (2.6–8.57)	1.74 (0.86–3.4)	0.12
	≥200	25	293	199	118	1	1	—

Weight for age (WFA)	Normal	35	249	180	116	1	1	—
	Moderate stunting	21	83	59	38	1.64 (0.95–2.82)	1.3 (0.6–2.71)	0.113
	Severe stunting	8	25	14	14	1.93 (0.89–4.1)	2.4 (0.80–7.3)	0.72

WHO	Stages 1 and 2	14	255	180	89	1	—	—
	Stages 3 and 4	50	102	73	79	5.70 (3.1–10.4)	1.44 (1.06–3.3)	0.032^*∗*^

Hemoglobin	>10 mg/dl	12	251	188	86	1	1	
	≤ 10 mg/dl	52	106	65	82	3.02 (2.18–4.77)	0.91 (0.42–1.9)	0.31

MUAC	>11.5 cm	12	261	179	74	1	1	—
	≤ 11.5 cm	40	108	74	94	7.9 (4.14–15.09)	4.436 (1.99–9.97)	0.001^*∗*^

## Data Availability

The data set for this research is in hand of main author and please ask him with reasonable request: fassikaw123@gmail.com.

## Abbreviations

IPT: Isoniazid preventive therapy
CPT: Cotrimoxazole preventive therapy
MUAC: Mid-upper-arm circumference
ART: Antiretroviral therapy, tuberculosis
WHO: World Health Organization
IDR: Incidence density rate
IQR: Interquartile range.
